# The meaning of losing a child in older adults: a qualitative study

**DOI:** 10.1186/s12877-021-02609-9

**Published:** 2021-11-19

**Authors:** Azade Safa, Mohsen Adib-Hajbaghery, Mahboubeh Rezaei, Marzieh Araban

**Affiliations:** 1 Trauma Nursing Research Center, Kashan University of Medical Sciences, 5th of Qotb –e Ravandi Blvd, P.O.Box: 8715981151, Kashan, Iran; 2 Department of Health Education and Promotion, School of Public Health, Ahvaz Jundishapur University of Medical Sciences, Ahvaz, Iran

**Keywords:** Meaning, Losing a child, Older adults, Content analysis, Qualitative study

## Abstract

**Background:**

After losing their child, elderly parents look for a meaning in this phenomenon. This meaning comes out from their experiences, and their responses to and actions in life are shaped based on this meaning. Therefore, this study was conducted with the aim of “understanding the meaning of losing a child in older adults.”

**Methods:**

This qualitative study was conducted using conventional content analysis method. Using semi-structured face-to-face interviews, data were collected from 15 older adults who had experienced of losing their adult child. Data analysis was performed according to the steps proposed by Graneheim and Lundman, 2004. To prove the trustworthiness of the data, credibility, dependability, confirmability and transferability were used.

**Results:**

The age range of participants was between 61 and 83 years and 73.3% of them were female. The two main categories of “tasting the bitter flavor of life” and “searching for a positive meaning in losing a child” together with the theme of “finding hope in the heart of darkness” were extracted from the participants’ experiences.

**Conclusions:**

Despite the grief of losing a child, which had cast a dark shadow over the parents’ lives, the child’s liberation from worldly sufferings, his/her presence in a better world, and being hopeful about the grace of God had caused the elderly parents to find hope in the heart of darkness. After identifying the parents with a deceased child, they should be helped through psychological counseling and care of the healthcare team so that they can adapt to this situation by finding a positive meaning in losing their child.

## Background

The loss of a child is one of the worst life events that can be experienced by parents and facing it can cause many physical, mental and emotional stresses [1]. Parents often feel helpless after this incident [2]. This psychological trauma has been compared in some studies to a hole in heart [3] or amputation [4]. Mourning the loss of a deceased child can involve a wide range of emotions. The grief caused by this loss is more intense and perdurable than other types of grief [5]. Naturally, after losing a loved one, the acute symptoms of grief subside gradually so that the grieving person can return to normal functioning within 1 to 2 months [5]. But, mourning the loss of a child can continue for a long time in the life of the parents, so that after such an event, a deep gap is made between some parents and the external world, affecting their interpersonal relationships and social functions and leading to their isolation [3].

Losing a child is a stressful event which may be experienced in old age. As the elderly population increases, more old people may experience the loss of a child [6]. Because of the internal factors associated with the aging process, they are more vulnerable to the effects of loss [7]. The coexistence of various losses in old age, such as retirement, children leaving home, and reduced health can jeopardize the older adults’ mental health [8], decreasing their vitality, self-confidence, and sense of usefulness [9]. The relationship between the older adults and their children varies in different cultures. In Eastern societies, children play a key role in the family structure and are responsible for looking after their old parents. In such cultures, children are considered as the most important source of support in old age [10, 11]. Therefore, losing a child for the older adults can have a special meaning.

After losing a child, parents look for a meaning and concept in this phenomenon which is derived from their experiences [12]. The meaning of losing a child in parents can be shaped in relation to themselves and their child or in interaction with others [13]. It is very important to know what the meaning of this phenomenon is in the mind of parents, as this meaning can function either as an incentive to help them cope with the crisis or an obstacle preventing them from adaptation to the new situation [14]. It is this meaning that gives shape to their responses and actions in life. Losing a child can even change the meaning of life. Accordingly, the challenge of finding a new meaning in life can lead to identity crisis in one’s life [14]. In a study, four themes extracted from the experiences of Chinese parents, who have lost their only child, were mental and physical health disorders, poor social interactions, loss of meaning in life, and lack of care and security [15]. The results of a study in South Africa also described the experiences of grief in old women after losing family members. The themes obtained in this study were being exposed to the loss of a large number of family members, the sadness of watching one’s death, being anxious about the imminent loss of other family members, prioritizing the feelings of family members, and spiritual and religious issues [16]. The results of another study showed in the experiences of parents a tension between meaninglessness and meaning in losing a child [12].

Understanding the meaning of losing a child, especially in the older adults, can show their understanding of the depth of the event and their views on this stressful event [17]. The meaning created by this phenomenon in the minds of parents can help members of the healthcare team to manage the situation, prevent its negative consequences and return these parents to normal life [18]. Previous studies in this area have been conducted using quantitative approach and in younger parents [19, 20], which cannot cover all aspects of this phenomenon. Therefore, the best method for the deep analysis of the meaning of losing a child is qualitative approach which has a more effective role in answering questions that involve human interpretations and mindsets and seeks the depth and complexity of phenomena [21]. Given that older adults constitute 10% of Iran’s population [22] and losing a child is strongly influenced by cultural and ethnic differences [5], no study has been conducted hitherto on Iranian older adults in this regard. Therefore, this question was raised in the mind of the researchers that “What is the meaning and concept of losing a child in elderly parents?” Answering this question provides a broader understanding of this phenomenon in different cultures. Given the vulnerability of the older adults, the unknown meaning of losing a child in them, and the lack of a related study on the Iranian older adults, this study was conducted with the aim of understanding the meaning of losing a child in the older adults.

## Methods

### Design

The present study is a qualitative research conducted using conventional content analysis. Content analysis is concerned with meanings, intentions, consequences and context. It can facilitate the understanding of the meaning of communications [23], discover people’s understanding of life phenomena, and interpret the content of subjective data [24]. In conventional content analysis, categories are extracted directly from the text of the data [25].

### Participants

The participants of the study were selected from the older adults who experienced the loss of their adult child in their old age. Inclusion criteria were 60-years-old or more, no cognitive problems such as Alzheimer’s, experience of losing an over 16 years old child, experience of losing a child in old age, at least 1 year after losing the child, consent to participate in the study and good ability to transfer experiences. Exclusion criterion was the participant’s decision to leave the research during the study. To consider the maximum variation, the participants with different characteristics of age, sex, financial and social status were included in the study.

### Data collection

Data were collected from October 2020 to June 2021, through semi-structured and in-depth face-to-face interviews with open-ended questions. Purposive sampling method was used in this study. It is a suitable sampling method for qualitative studies where the researcher tries to recruit informants who have the best knowledge with regard to the investigated phenomenon [26]. The researcher found the first two participants by searching among colleagues and friends and, then, according to the data obtained from them, the next four participants were selected from among the older adults who had been registered at three primary health care centers (PHCC) in Kashan and Isfahan cities. There are several PHCCs in all rural and urban parts of Iran. PHCCs are affiliated with the Ministry of Health and Medical Education, and each center registers all older adults living in its area and covers them for primary healthcare services. Some of the participants were also older adults with similar experiences who were introduced to the researcher by the former participants. For the interview arrangement, the participant was contacted by telephone. After introducing herself and explaining the purpose of the research, the researcher determined the time and place of the interview based on the desire of the participant if s/he agreed to participate in the study. In this study, 11 interviews were conducted at the participants’ home, two interviews at the nursing school, one in the park, and one at the grave of the deceased child. The interviews were conducted in a calm environment and were recorded using a digital MP3 recorder with the permission of the participants. During the interviews, the participants were asked these key questions: “Describe your experience of losing your child” and “What does losing a child mean to you?” During the interviews, probing questions such as “explain more,” “make your point clearer,” and “give an example” were asked to encourage the participant and gain more in-depth information. The interviews lasted between 42 and 72 min, and the data collection process continued until no new conceptual codes were emerged. In three cases, because of fatigue of the participant, the interview was conducted in two sessions. Two eligible elderly parents refused to participate in the study as they were busy.

### Data analysis

Data analysis began after the first interview and continued alongside data collection. This aided the reciprocal process between conceptualization and data collection. The data analysis process was performed according to the steps proposed by Graneheim and Lundman [24]. Thus, after the end of each interview, the verbatim transcription of its content was performed as soon as possible. The text of the typed interviews was transferred to MAXQDA software version 12 and the data were managed using this software. The transcripts of the interviews were read several times by the main researcher (PhD student) to gain a general understanding of them. First, the units of analysis were identified. The whole text of each interview was considered as a unit of analysis. Then, the words, sentences or paragraphs were considered as meaning units. Based on their contents, these units were put together and formed condensed meaning units. Condensed meaning units were abstracted, labeled and converted into codes. The codes were made as the actual words of the participants (invivo coding) or as the words that were the researcher’s general understanding of their speech (invitro coding). The codes were compared with each other in terms of similarities and differences and were classified into 7 subcategories and 2 main categories. Finally, by comparing the categories with each other and deep reflection, the content hidden in the data was introduced as the theme “finding hope in the heart of darkness”.

### Trustworthiness

In order to determine the trustworthiness of the research data, four criteria proposed by Guba and Lincoln (1985) were used [27]. Prolonged engagement, and appropriate interaction with participants helped to increase the credibility of the findings. Moreover, member checking was used to increase the credibility of the analysis. The full transcribed texts of some interviews along with the codes extracted from them were returned to the participants in order to check whether they are the resonance of their experiences or not.

In order to increase the dependability of the data, the codes extracted from each interview were peer checked by the colleagues and experts of this field. The use of sufficient and appropriate samples with maximum variation strengthened the confirmability of the data. Moreover, through providing direct quotes, the researcher tried to ensure the transferability of the data.

### Ethical consideration

After approving the study protocol in the ethics committee of Kashan University of Medical Sciences with the ethics code of IR.KAUMS.NUHEPM.REC.1399.049, a letter of introduction was obtained from the research vice chancellor of the university. The project was found to be in accordance to the ethical principles and the national norms and standards for conducting medical research in Iran. Before the interview, the objectives of the study were explained to the participants and written and informed consent was obtained from them for participating in the research and recording the interviews. Written consent was obtained separately for each individual interview. The participants were assured that their information would remain confidential. They would also be provided with research results if they wished. Given the concurrency of data collection and COVID-19 pandemic, all health protocols were observed during the interview.

## Results

In the present study, 15 eligible participants were interviewed. The age range of participants was between 61 and 83 years and 73.3% of them were female. Ten of the deceased children were male and five others were female. One to 10 years had passed from losing the child (Table 1).

**Table 1:** Demographic characteristics of the participants

Participant	Gender	Age (year)	Marital status	Occupation	Level of education	Cause of child death
P #1	Female	75	Widow	Retired	Diploma	Accident
P #2	Female	82	Widow	Housewife	Uneducated	Cancer
P #3	Male	62	Married	Self-employed	Diploma	Cancer
P #4	Female	63	Married	Housewife	Diploma	Cancer
P #5	Female	64	Married	Retired	Academic	Burn
P #6	Female	65	Married	Housewife	Diploma	Accident
P #7	Male	63	Married	Retired	Diploma	Martyrdom
P #8	Female	83	Widow	Housewife	Uneducated	Overdose
P #9	Female	69	Married	Retired	Academic	Accident
P #10	Male	61	Married	Self-employed	Primary school	Covid-19
P #11	Female	65	Married	Housewife	Primary school	Poisoning
P #12	Female	71	Widow	Housewife	Primary school	Drowning
P #13	Female	65	Married	Housewife	Academic	Covid-19
P #14	Male	78	Married	Self-employed	Uneducated	Kidney disease
P #15	Female	72	Widow	Housewife	Academic	Burn

Two main categories and 7 sub-categories were emerged from 392 primary codes. In data analysis, the two main categories of “tasting the bitter flavor of life” and “searching for a positive meaning in losing a child” together with the theme of “finding hope in the heart of darkness” were extracted from the participants’ experiences (Table 2).

**Table 2:** The Categories extracted from the meaning of losing a child in elderly parents

Theme	Categories	Subcategories	Examples of initial coding
Finding hope in the heart of darkness	Tasting the bitter flavor of life	Difficulty in enduring grief	Losing a child as one of the most difficult tragedies Endless grief An unforgettable sorrow
		Lack of understanding of tragedy by others	Ignorance of others about the situation of the parents Lack of understanding of the problem by friends The unthinkable loss of a child
		Separation of a part of your body	Losing half of the mother’s being Heartache Removing one part of the mother’s being
	Searching for a positive meaning in losing a child	Liberation from worldly sufferings	End of the child’s sufferings The child’s release from the material world The child’s release from the disease
		Joining the beauties	To begin a new life The child’s joining to light Going to a better world
		Divine Providence	God’s will in losing one’s child Losing a child as a divine destiny End of the child’s life determined by God’s will
		Divine Trial	Exhibition of God’s power The test of patience by God Passing a really hard divine trial

### Finding hope in the heart of darkness

The grief of losing a child was a heavy sorrow in the heart of parents that had darkened their lives. However, the child’s liberation from the pains and sufferings of the world, their presence in a better world, and being successful in the divine trial caused the parents to find hope in the heart of darkness.

#### Tasting the bitter flavor of life

The parents described the loss of their child as the most tragic event in their lives. This event changed their lives to the extent that after losing their child they no longer experienced happiness. This category included three subcategories of “difficulty in enduring grief,” “lack of understanding of tragedy by others,” and “separation of a part of your body”.

##### Difficulty in enduring grief

In this subcategory, the participants referred to the difficulty in enduring the grief of losing a child. Losing their child, the parents had lost their dreams for their child and this hurt their hearts. *“I used to be very happy; but after that I see sorrow in my face when I look in the mirror. I had lots of dreams for him; all those dreams have gone. Now, I have pain, a pain in my heart.”* (P #9)They also maintained that their lives never went back to the normal life they had before this tragedy. The parents described this grief as a heavy burden they should carry with them throughout their lives.

*“... I will bear a great sorrow for the rest of my life. This is a really incurable pain.*” (P #6) Tolerating this grief could gradually cause burnout in the parents.

##### Lack of understanding of tragedy by others

The statements of the participants showed that in their view, losing a child is so heart-rending that others are unable to comprehend it. This issue caused some participants to talk less with others in this regard, cut off communication with others and refuse to receive support from others.

*“You can't talk much about this to anyone; they can't understand it, because the nature of this problem is different from other problems.”* (P #10)

Some parents also stated that only those who have similar experiences can understand it as it is a really heartbreaking phenomenon. *“I don’t think anyone can understand me until they experience the tragedy of child losing. My heart is always filled with sorrow. Who knows what happens to a mother?”* (P #12).

##### Separation of a part of your body

According to most mothers, the child was likened to a part of the mother’s body, especially her heart and, thus, a part of the mother’s being had been separated from her by losing her child. This loss was described as a never-healed wound.

*“My child was part of my body. Losing him was like a wound which never will be healed.”* (P #11)

One of the participants likened the grief of losing their child to heartache. Many parents developed heart disease after this incident, which they attributed to grief caused by losing their child.

*“It is really unbelievable. After losing my child, I felt that one part of my heart was removed or I had no heart at all. I didn't think I would be alive after he left. I'm suffering from heart disease after that.*” (P #5)

#### Searching for a positive meaning in losing a child

The parents of this study sought positive meanings in losing their child. Based on the experiences of the parents, positive meanings in losing a child included “liberation from worldly sufferings”, “joining the beauties”, “divine providence”, and “divine trial”. Finding this meaning was an incentive for them to continue living without their children.

##### Liberation from worldly sufferings

This subcategory emerged from the experiences of the participants whose child had died because of an incurable disease. For them, liberation from worldly sufferings meant relief from the pain and suffering their child was going through. These parents witnessed their children’s physical and mental pain during their illness, and believed that death had relieved them of these sufferings.

*“I woke up at nights by the sound of her moaning; many nights she suffered until morning and got many nosebleeds. The memory of these still lingers in my mind. She left and was relieved of these pains.”* (P #4)

The process of losing a child was formed in these parents over time and, thus, finding a meaning in this incidence helped parents to better cope with the loss of their child. Another participant stated: *“I tell myself that he is now relieved of his suffering; he did not have a good life in this world; may he have a better one in the other world.”* (P #8).

##### Joining the beauties

Joining the beauties was, from the participants’ point of view, the child’s living in a better world. All parents of the study believed in afterlife and even after losing their child tried to find out more about it. They believed that their child had gone to a far more beautiful and better world to continue living. The hope of seeing each other again was, thus, an incentive for the parents to continue living.

*“The only thing that makes me sad is that I miss him; but, I know he's fine in the other world. My son has joined the light and we'll see each other very soon.”* (P #1)

The parents also had recurrently dreamed of their child in a beautiful place, and this made them less impatient and calmer.

*“One day I asked God ‘show me that my child has a good place and then I'll calm down and won't cry anymore.’ That night I had a dream. He was in a beautiful garden; an indescribable beautiful one. He said ‘don’t worry about me mom; my place is good.”* (P #13)

##### Divine Providence

The parents considered the will and power of God important in losing their child and believed that this event had been predestined for them. Some of the parents complained to God in the first months after losing their child but, eventually, spirituality and religious beliefs caused them to find a meaning in this event.

*“...I realized that when something is going to happen, it will happen in its own time. We should not be too happy or sad about things. Everyone's lifetime has been predetermined by God. My child had also a predestined lifetime.”* (P #4)

*“There are a series of things on which we have no control. Destiny cannot be stopped. Every human being has a destiny. The loss of a child has also been determined in my destiny. We also surrender to whatever God wills.”* (P #14)

##### Divine trial

The parents considered losing a child as a means of divine trial, which they could pass by enduring and patience.

*“God tests everyone in the world. I was also tested by the loss of my child. God wants to see if I am ungrateful or I can bear it! However, patience is also granted to you by God, otherwise one cannot live without one's child.”* (P #2)

This meaning provided the parents with an incentive to bear the grief of loss. Some parents, especially those who had lost their children for lofty purposes such as defending the homeland, considered themselves successful in this trial and believed that they would receive God’s grace and reward.

*“The martyrdom of a child is a crown of honor on the head of parents who think their child has been useful for the country, for the safety of his people. In fact, we have passed God's trial successfully and God will reward us for it.”* (P #7)

## Discussion

This qualitative study explored the meaning of losing a child in the older adults. After losing their child, the elderly parents sought a meaning for this phenomenon. Based on their experiences, the theme of “finding hope in the heart of darkness” appeared in two categories of “tasting the bitter flavor of life” and “searching for a positive meaning in losing a child”. As parents continued to live without their children, they found new meanings which could help them cope with the grief of losing their child. Despite grief of losing a child that had cast a dark shadow on the parents’ lives, the child’s liberation from worldly sufferings, their presence in a better world, passing the divine trial and being hopeful to God’s mercy, had caused the parents to find hope in the heart of darkness. Finding positive meanings helped them cope with the loss of their child and prevented negative consequences. By contrast, finding negative meanings in losing a child could also be a threat to their mental and physical health.

In this study, the older adults believed that losing their child had been the most bitter and difficult loss in their lives. They were so sad after losing their child that they did not feel any inner joy after that. It was found in a study that among the deaths of family members, losing an adult child is the most tragic event for an older adult [16]. In the study of Berrett-Abebe et al. [28], “the experience of a sad life” was one of the themes obtained from mothers whose child had died because of cancer. This bitter experience might be attributable to the fact that older parents had spent more time with their children and had stronger bonds with them. Then, they suffer more after losing them.

From the parents’ point of view, their grief and suffering was not understood by others. This view can cause serious harms to the parents when they need support. They might hide their feelings and limit their communication with others which could consequently increase the likelihood of social isolation and lead to the risk of anxiety disorders and depression in them [29]. According to the results of a study, the need to be understood, being supported by the family and health team and sharing one’s experiences are essential in coping with this phenomenon [30].

In this study, most parents, especially mothers, likened their child to a part of their being that was separated from them by death. After losing their child, they had heart problem caused by the grief of losing their child. It was shown in a study that the consequences of losing a child in mothers were more severe than fathers [29]. Given the vulnerability of older adults and the increased rate of heart disease in them, this phenomenon can have serious consequences and requires further investigation and follow-up in these people.

Most parents sought to find a positive meaning in losing their child. The parents, who had lost their child by a chronic disease such as cancer, had seen their child suffering from the time of diagnosis until death and, thus, death was considered by them a relief from the pains of the illness. Investigating the response of mothers of children with cancer, Gerrish et al. [31] depicted that these mothers knew that their child would soon die, and observing the child’s suffering was a form of torture for both mother and child. Thus, it seems logical for parents to consider the loss of their child as a release from this torture. In another study, losing a child for some parents meant relief from physical and mental suffering [14]. For the parents who believed in afterlife, the loss of their child meant joining the beauties of that world. They believed that their child had been transferred to a safe and beautiful place to live. This belief was a means of peace for them. However, it was reported in a study that the search for meaning after dealing with death was a bothering experience [32]. In another study on younger parents, one-fourth of the participants stated that there is no positive meaning in a terrible event such as losing a child [14]. Such controversies might be attributable to the differences in the age and culture of the participants.

Because of adherence to spirituality and having religious beliefs, the parents of this study believed that the loss of their child had been the work of providence and God’s will. Faith in God and trusting Him had helped them manage the situation. Being successful in divine trial and having hope for God’s reward were important motivations for enduring the hardships of losing a child and continuing to live. In the study of Lekalakala-Mokgele et al. (2018), spiritual and religious issues played an important role in the adaptation of old women to the loss of their family members [16]. But, according to another study, there was no significant relationship between spirituality, resilience, and adaptation after major psychological trauma [33]. It seems that spirituality and religion play a significantly positive role in dealing with stressful events of life, as people with better spiritual beliefs have been able to accept the loss of their child by constructing a meaning in this event. Reliance on spirituality increases in old age and, using their religious beliefs, the older adults strive to be successful in the divine trial and be worthy of God’s reward. Therefore, relying on these beliefs, nurses can help parents, especially older adults, to deal with their identity crisis after a loss. In general, finding a meaning in life by this group of people will promote the mental health of parents and their quality of life.

Loss of a child, especially in elderly parents, can cause anxiety, depression, and changes in social interaction [5]. How to deal with a phenomenon is formed through the meaning of that phenomenon created in one’s mind. The meaning found by parents in the loss of their child is very important as it can be the root of many of their problems or protect them from problems. Finding meaning in the loss of their child, parents can revive their life [34]. Given the increasing number of older people around the world, finding a positive meaning in losing a child can help this vulnerable group reduce the predicted negative consequences and revive their life [14, 34]. Members of the healthcare team, especially nurses working in the areas of gerontological and psychological care, are responsible for identifying, supporting and advising this group of people. These parents can be supported through proper communication, active listening, understanding of their feelings, regular follow-up, and physical and mental examinations. Therefore, it is recommended that parents whose child had died be identified through their health records in health centers. Doing so, they can receive nursing care and psychological counseling to adapt to this event by founding a positive meaning in it.

### Strengths of the study

This was the first study which investigated the meaning of losing a child in Iranian older adults. It presented the meaning of losing a child in older adults by pointing out its dimensions and revealed the categories which were less addressed in previous studies. These findings might be used as a basis for developing health metric assessment tools and evidence-based elderly health promotion programs by authorities. The results of this study can increase the knowledge of healthcare team about this phenomenon and help them provide better care for these parents.

### Limitation of the study

Most of our participants were elderly mothers. Thus, it is suggested that further studies focus more on fathers’ experiences to determine whether there is a difference between mothers and fathers in terms of the meaning of losing their child.

## Conclusion

According to the statements of the participants, the theme of “finding hope in the heart of darkness” appeared in two categories of “tasting the bitter flavor of life” and “searching for a positive meaning in losing a child”. Despite the fact that this grief had cast a dark shadow on the parents’ lives, the child’s liberation from worldly sufferings, their presence in a better world, passing the divine trial and being hopeful to God’s mercy, had caused the parents to find hope in the heart of darkness. Finding a positive meaning in losing a child can make parents adapt to this tragedy, so that despite this loss, they continue to live purposefully. Accordingly, healthcare providers and counselors can help these parents to adapt to this loss through finding a positive meaning in it.

## Data Availability

The dataset collected and analyzed in the current study are not publicly available due to ethical restrictions to maintain the participants’ anonymity. The corresponding author can be contacted on reasonable requests regarding the dataset.

## Abbreviations

PHCC: Primary Health Care Center
P: Participant

## References

[1] Bratt AS, Stenström U, Rennemark M (2018). Exploring the most important negative life events in older adults bereaved of child, spouse, or both. Omega (Westport).

[2] Shifa GT, Ahmed AA, Yalew AW (2018). The relationship between under-five child death and maternal mental distress in Gamo Gofa zone, southern Ethiopia: a community based comparative cross-sectional study. BMC Womens Health.

[3] Snaman JM, Kaye EC, Torres C, Gibson DV, Baker JN (2016). Helping parents live with the hole in their heart: the role of health care providers and institutions in the bereaved parents’ grief journeys. Cancer..

[4] Toller PW (2011). Bereaved parents’ experiences of supportive and unsupportive communication. South Commun J.

[5] Gijzen S, L'Hoir MP, Boere-Boonekamp MM, Need A (2016). How do parents experience support after the death of their child?. BMC Pediatr.

[6] Dixon A (2021). The United Nations decade of healthy ageing requires concerted global action. Nat Aging.

[7] Monk TH, Pfoff MK, Zarotney JR (2013). Depression in the spousally bereaved elderly: correlations with subjective sleep measures. Depress Res Treat.

[8] Gao M, Li Y, Zhang S, Gu L, Zhang J, Li Z (2017). Does an empty nest affect elders’ health? Empirical evidence from China. Int J Environ Res Public Health.

[9] Parkar SR (2015). Elderly mental health: needs. Mens Sana Monogr.

[10] Nouri A, Farsi S (2018). Expectations of institutionalized elderly from their children. Salmand: Iranian J Ageing.

[11] Mughal S, Siddiqui WJ (2019). Grief reaction. StatPearls.

[12] Titus B, de Souza R (2011). Finding meaning in the loss of a child: journeys of chaos and quest. Health Commun.

[13] Tseng YF, Hsu MT, Hsieh YT, Cheng HR (2018). The meaning of rituals after a stillbirth: a qualitative study of mothers with a stillborn baby. J Clin Nurs.

[14] Bogensperger J, Lueger-Schuster B. Losing a child: finding meaning in bereavement. Eur J Psychotraumatol. 2014;5. 10.3402/ejpt.v5.22910. 10.3402/ejpt.v5.22910 PMC3972418 24765248

[15] Wang N, Hu Q (2021). “It is not simply the loss of a child”: the challenges facing parents who have lost their only child in post-reproductive age in China. Death Stud.

[16] Lekalakala-Mokgele E (2018). Death and dying: elderly persons’experiences of grief over the loss of family members. S Afr Fam Pract.

[17] Floyd FJ, Mailick Seltzer M, Greenberg JS, Song J (2013). Parental bereavement during mid-to-later life: pre- to postbereavement functioning and intrapersonal resources for coping. Psychol Aging.

[18] Judith S (2014). The role of nursing in health care. Rev Bras Enferm.

[19] Pohlkamp L, Kreicbergs U, Sveen J (2019). Bereaved mothers’ and fathers’ prolonged grief and psychological health 1-5 years after loss - a nationwide study. Psychooncology..

[20] Lykke C, Ekholm O, Schmiegelow K, Olsen M, Sjøgren P (2019). Anxiety and depression in bereaved parents after losing a child due to life-limiting diagnoses: a Danish Nationwide Questionnaire Survey. J Pain Symptom Manag.

[21] Bengtsson M (2016). How to plan and perform a qualitative study using content analysis. Nursing Plus Open.

[22] Statistical Center of Iran (2018). Population and housing census.

[23] Cavanagh S (1997). Content analysis: concepts, methods and applications. Nurse Res.

[24] Graneheim UH, Lundman B (2004). Qualitative content analysis in nursing research: concepts, procedures and measures to achieve trustworthiness. Nurse Educ Today.

[25] Erlingsson C, Brysiewicz P (2017). A hands-on guide to doing content analysis. Afr J Emerg Med.

[26] Elo S, Kaariainen M, Kanste O, Polkki T, Utriainen K, Kyngas H (2014). Qualitative content analysis: a focus on trustworthiness. SAGE Open.

[27] Lincoln, YS, Guba EG. Naturalistic Inquiry. Newbury Park: Sage Publications, 1985.

[28] Berrett-Abebe J, Levin-Russman E, Gioiella ME, Adams JM (2017). Parental experiences with a hospital-based bereavement program following the loss of a child to cancer. Palliat Support Care.

[29] Lee C, Glei DA, Weinstein M, Goldman N (2014). Death of a child and parental wellbeing in old age: evidence from Taiwan. Soc Sci Med.

[30] Price JE, Jones AM (2015). Living through the life-altering loss of a child: a narrative review. Issues Compr Pediatr Nurs.

[31] Gerrish NJ, Bailey S (2020). Maternal grief: a qualitative investigation of mothers’ responses to the death of a child from cancer. Omega (Westport)..

[32] Linley PA, Joseph S (2011). Meaning in life and posttraumatic growth. J Loss Trauma.

[33] Bhattarai M, Maneewat K, Sae-Sia W (2018). Psychosocial factors affecting resilience in Nepalese individuals with earthquake-related spinal cord injury: a cross-sectional study. BMC Psychiatry.

[34] Barak A, Leichtentritt RD (2015). Ideological meaning making after the loss of a child: the case of Israeli bereaved parents. Death Stud..

